# Emotional Intelligence and Leadership in Nursing Management: The Role of Hierarchical Context in Shaping Leadership Behaviour

**DOI:** 10.1111/jan.70680

**Published:** 2026-07-24

**Authors:** Paschalina Ntotsi, Mary Gouva, Maria Lavdaniti, Evgenia Minasidou

**Affiliations:** ^1^ General Hospital of Thessaloniki ‘G. Gennimatas’ Thessaloniki Greece; ^2^ Department of Nursing University of Ioannina Ioannina Greece; ^3^ Department of Nursing International Hellenic University Thessaloniki Greece

**Keywords:** emotional intelligence, hierarchical context, leadership styles, nurse managers, nursing leadership, organisational factors

## Abstract

**Aim:**

To examine the relationship between emotional intelligence (EI) and leadership style among nurse managers, and how the relationship is shaped by professional experience, hierarchical position, and healthcare facility size. The study explores whether EI functions as a conditional mechanism linking experience to leadership across hierarchical levels.

**Design:**

A cross‐sectional quantitative study.

**Methods:**

A total of 1243 nurse managers from public healthcare facilities participated in a survey conducted between November 2022 and January 2023. Emotional intelligence was measured using the Trait Emotional Intelligence Questionnaire–Short Form (TEIQue‐SF), and leadership style using the Multifactor Leadership Questionnaire (MLQ). Differences across hierarchical levels were examined using repeated measures ANOVA. A conditional process analysis (PROCESS Model 59) tested the mediating role of EI and the moderating effect of hierarchical position, controlling for gender and facility size.

**Results:**

Transformational and transactional leadership were more pronounced among higher‐level managers, whereas passive leadership was more prevalent among front‐line managers. Emotional intelligence was positively associated with transformational and transactional leadership and negatively associated with passive leadership. EI demonstrated a conditional and partial mediating role in the relationship between professional experience and leadership, with effects varying across hierarchical levels. Hierarchical position moderated both the relationship between experience and EI and the association between EI and transformational leadership. Managers in larger healthcare facilities reported higher EI and lower passive leadership.

**Conclusion:**

Leadership in nursing management is shaped by the interaction between emotional competencies and organisational structure. Emotional intelligence operates as a context‐dependent capacity, whose development and expression are conditioned by hierarchical position and organisational environment.

**Impact:**

This study highlights that emotionally intelligent leadership is not solely an individual attribute but is structurally enabled or constrained. Supporting nurse managers requires organisational interventions, including supervisory time, mentorship, and conditions that sustain emotional regulation and reflective capacity.

**Patient or Public Contribution:**

No patient or public contribution.

## 1 Introduction

Nursing leadership is a key determinant of care quality, staff performance, and organisational functioning. Beyond clinical expertise, nurse managers are required to coordinate teams, regulate emotionally charged interactions, and make decisions under pressure. These demands vary across hierarchical levels, ranging from strategic oversight to direct staff supervision and patient care coordination (Stordeur et al. 2000). Previous research has demonstrated that transformational leadership in nursing is associated with improved staff satisfaction, patient safety outcomes, and organisational functioning (Boamah et al. 2018).

Emotional intelligence (EI), defined as the capacity to perceive, understand, and manage emotions (Goleman 1998), has been widely recognised as a central leadership competency in healthcare. Higher EI among nurse managers has been associated with improved communication, conflict management, and relational leadership behaviours (Gardner and Stough 2002; Spano‐Szekely et al. 2016). However, growing evidence suggests that EI does not operate uniformly across organisational contexts. Rather than functioning solely as an individual skill, its expression may be shaped by professional experience, hierarchical position, and structural demands.

Leadership practices differ across hierarchical levels, reflecting variations in responsibility, autonomy, and exposure to emotional labour (Wong et al. 2013). A growing body of evidence further suggests that leadership styles in nursing are closely associated with workforce wellbeing, organisational climate, and patient‐related outcomes (Cummings et al. 2018). Despite this, the extent to which hierarchical position conditions the relationship between EI and leadership style remains insufficiently understood. Similarly, the role of healthcare facility size in shaping emotional and leadership demands has received limited empirical attention, despite its potential influence on workload, relational complexity, and managerial expectations.

From an organisational perspective, leadership behaviour does not emerge solely from individual traits, but from the interaction between personal capacities and structural working conditions. Role theory suggests that hierarchical positions involve different expectations, responsibilities, and emotional demands, which may shape how leadership competencies are enacted in practice (Kahn et al. 1964; Heller et al. 2023). Similarly, job demands–resources perspectives propose that emotionally demanding work environments can either facilitate or constrain the development and expression of adaptive leadership capacities depending on the availability of organisational resources, support, and autonomy (Bakker and Demerouti 2007). High job demands combined with insufficient organisational resources have been associated with burnout and reduced engagement among nurses, particularly in complex clinical environments (Montgomery et al. 2015). Within nursing management, front‐line managers are often exposed to sustained emotional labour, high workload demands, and competing clinical and administrative pressures, whereas higher‐level managers may have greater access to strategic control, organisational resources, and reflective decision‐making opportunities (Wong et al. 2013; Cranage and Foster 2022). These structural differences may influence not only leadership behaviour itself, but also the extent to which emotional intelligence can be translated into effective leadership practice. Accordingly, emotional intelligence in nursing leadership may be better understood not simply as an individual competency, but as a contextually enacted capacity whose expression depends on organisational and hierarchical conditions.

Within this framework, leadership in nursing management can be understood as the outcome of an interaction between individual psychological capacities and organisational structure. Nurse managers operate in emotionally demanding environments, where the ability to recognise, interpret, and regulate emotional cues is essential for coordinating teams, managing conflict, and maintaining care quality (Prezerakos 2018). EI is therefore not merely an interpersonal attribute, but a context‐sensitive capacity whose effectiveness may depend on the conditions under which leadership is enacted.

Consistent evidence links higher levels of EI to transformational leadership, characterised by inspirational motivation, intellectual stimulation, and relational attunement (Bass and Riggio 2006; Spano‐Szekely et al. 2016; Tyczkowski et al. 2015). Leaders with higher EI are more capable of mobilising shared purpose and navigating emotionally complex situations. However, findings regarding transactional and passive (laissez‐faire) leadership remain less consistent (Harms and Credé 2010). While transactional leadership may benefit from emotional regulation in performance monitoring, it does not necessarily require deep relational engagement. Passive leadership, by contrast, has been associated with lower EI and poorer organisational outcomes (Hinkin and Schriesheim 2008), although its emergence may also reflect structural constraints rather than individual deficits.

Professional experience represents a further dimension through which EI may develop. Exposure to emotionally demanding clinical and managerial situations can enhance emotional competencies over time (Zeidner et al. 2009; Cherry et al. 2014). However, this relationship may not be linear, as prolonged exposure to high‐intensity environments without adequate organisational support may also lead to emotional depletion.

Hierarchical position may further condition both the development and the expression of EI. Top‐level managers primarily engage in strategic and system‐level functions, while middle‐level managers mediate between organisational demands and frontline realities. Front‐line managers, in contrast, operate at the intersection of direct care and administrative responsibility, often under conditions of heightened emotional and operational pressure (Cranage and Foster 2022). These differences suggest that both EI and leadership style may vary systematically across hierarchical levels.

The present study examines how professional experience, emotional intelligence, hierarchical position, and healthcare facility size jointly shape leadership style among nurse managers. It further investigates whether EI demonstrated conditional indirect associations between professional experience and leadership style across hierarchical levels, and whether these pathways are moderated by hierarchical position. By analysing these factors simultaneously, the study seeks to clarify the organisational conditions under which emotional competencies translate into effective leadership.

### 1.1 Hypotheses

#### 1.1.1 Direct Associations Between Emotional Intelligence and Leadership Style

*Emotional intelligence is positively associated with transformational leadership*.

*Emotional intelligence is negatively associated with passive leadership*.

*Emotional intelligence is positively associated with transactional leadership*.

#### 1.1.2 Associations Between Experience, Hierarchy, and Emotional Intelligence

*The association between professional experience and emotional intelligence varies across organisational contexts and hierarchical roles*.

*Hierarchical level is associated with emotional intelligence*.

*Hierarchical level is associated with leadership style*.

#### 1.1.3 Conditional (Moderation) Effects of Hierarchy

*Hierarchical level moderates the relationship between professional experience and emotional intelligence*.

*Hierarchical level moderates the relationship between emotional intelligence and leadership style*.

#### 1.1.4 Conditional Process (Mediated and Contextual Effects)

*Emotional intelligence demonstrates a conditional mediating role in the relationship between professional experience and leadership style*.

#### 1.1.5 Organisational Context (Facility Size)

*Healthcare facility size is positively associated with emotional intelligence*.

*Healthcare facility size is associated with leadership style*.

## 2 Methods

### 2.1 Study Design and Participants

A cross‐sectional study was conducted among nurse managers employed in public healthcare facilities. Data were collected between November 2022 and January 2023. Of the initial sample of 1266 respondents, 19 were excluded due to missing demographic information, and four were excluded due to incomplete or non‐classifiable responses on gender. The final analytic sample comprised 1243 participants (98.2% of the initial respondents), representing nurse managers across three hierarchical levels: top‐level, middle‐level, and front‐line managers.

To account for organisational variation, healthcare facilities were categorised as large (major urban hospitals) or small (regional hospitals or primary care units). This distinction reflects differences in administrative complexity, staffing structures, and care delivery volume, which may influence managerial roles and demands. The study was reported in accordance with the Strengthening the Reporting of Observational Studies in Epidemiology (STROBE) guidelines for cross‐sectional studies.

### 2.2 Measures

#### 2.2.1 Sociodemographic Questionnaire

Participants provided information on gender, age, professional experience (years in practice), hierarchical level, and healthcare facility size.

#### 2.2.2 Emotional Intelligence

Emotional intelligence was assessed using the Trait Emotional Intelligence Questionnaire–Short Form (TEIQue‐SF), a 30‐item instrument rated on a 7‐point Likert scale. The validated local adaptation of the scale has demonstrated satisfactory validity and reliability (Stamatopoulou et al. 2016). A total EI score was calculated as the mean of all items, following reverse scoring where appropriate, with higher scores indicating greater emotional intelligence.

#### 2.2.3 Leadership Style

Leadership style was measured using the Multifactor Leadership Questionnaire (MLQ), which assesses transformational, transactional, and passive‐avoidant leadership. Subscale scores were computed in accordance with the recommendations of Bass and Avolio (1990). Internal consistency of all subscales was examined prior to analysis.

The Management‐by‐Exception subscale demonstrated poor internal consistency (Cronbach's *α* = 0.31) and was therefore excluded from further analyses. This decision is consistent with prior research indicating that this dimension may exhibit measurement instability in healthcare leadership contexts (Hinkin and Schriesheim 2008).

In line with the theoretical structure of the MLQ, leadership dimensions were operationalised as follows: transformational leadership was calculated as the mean of Individualised Consideration (IC), Idealised Influence (II), Inspirational Motivation (IM), and Intellectual Stimulation (IS); transactional leadership was represented by the Contingent Reward (CR) subscale; and passive leadership was represented by the Laissez‐Faire (LF) subscale.

This scoring approach is consistent with previous leadership research and enhances the interpretability of the constructs.

However, because transactional leadership was operationalised primarily through the Contingent Reward dimension following the exclusion of Management‐by‐Exception, the construct should be interpreted as reflecting a narrower representation of transactional leadership behaviours. In addition, the internal consistency of the Contingent Reward subscale was modest, and findings related to transactional leadership should therefore be interpreted with caution.

### 2.3 Statistical Analysis

Analyses were conducted using SPSS (version 21) and R (R Core Team 2021), incorporating PROCESS Model 59 (Hayes 2017). Preliminary analyses included descriptive statistics and internal consistency assessment of all study variables.

Differences in leadership profile patterns across hierarchical levels were examined using repeated measures ANOVA. This analysis was used descriptively to compare the relative prominence of different leadership dimensions within the same participants across hierarchical groups, rather than to imply that the leadership styles represent repeated measurements of a single construct.

The primary analysis employed a conditional process model (moderated mediation), enabling the simultaneous examination of indirect and interaction effects. Specifically, emotional intelligence was modelled as a mediator of the association between professional experience and leadership style, while hierarchical level was specified as a moderator of both (a) the relationship between professional experience and emotional intelligence and (b) the relationship between emotional intelligence and leadership style.

Hierarchical level was entered as a set of dummy variables, with front‐line managers serving as the reference category. Facility size and gender were included as covariates in all models. Indirect effects were estimated using 5000 bootstrap samples to obtain bias‐corrected 95% confidence intervals. Where significant interaction effects were identified, simple slopes analyses were conducted to facilitate interpretation.

This analytical approach allows for the integrated examination of emotional, experiential, and structural influences on leadership outcomes, providing a theoretically grounded framework for understanding variation across managerial roles.

### 2.4 Ethical Considerations

The study was conducted within the framework of a doctoral research project and followed the required institutional and administrative approval procedures of the participating healthcare organisations prior to data collection. Formal approval was subsequently obtained from the relevant Research Ethics and Deontology Committee (Approval No: 59/2024; Meeting 4/27‐05‐2024; Decision date: 15/07/2024). All participants were informed about the purpose of the study and provided informed consent prior to participation. Participation was voluntary, and anonymity and confidentiality were ensured throughout the study.

## 3 Results

### 3.1 Sample Demographics

Table 1 summarises the sociodemographic characteristics of the 1243 respondents. Post hoc comparisons using the Least Significant Difference (LSD) test indicated that top‐level managers were significantly older and had greater professional experience compared to those in lower hierarchical positions. In contrast, front‐line managers constituted the youngest subgroup and reported the lowest levels of professional experience.

**TABLE 1:** Sociodemographic characteristics of the sample.

	Total	Hierarchy level			*p*
		Top‐level	Middle‐level	Front‐line	
Gender					
Female	1057	106	432	519	0.837^a^
Male	186	20	79	87	
Facility size					
Small	594	69	239	286	0.252^a^
Large	649	57	272	320	
Age (years), mean (SD)	52.6 (5.9)	54.8 (5.4)	53.6 (5.6)	51.2 (5.9)	< 0.001^b^
Professional experience (years), mean (SD)	28.4 (6.7)	32.5 (6.1)	29.8 (6.2)	26.4 (6.5)	< 0.001^b^

^a^ Chi square test of independence.

^b^ One‐way analysis of variance.

### 3.2 Leadership Styles Scores

Of the seven leadership subscales, six demonstrated modest to excellent internal consistency (Table 2). In contrast, the Management‐by‐Exception subscale showed poor internal consistency (Cronbach's *α* = 0.31) and was therefore excluded from subsequent analyses. This decision is consistent with prior research indicating that the Management‐by‐Exception dimension often exhibits measurement instability and conceptual ambiguity, particularly in nursing leadership contexts (Hinkin and Schriesheim 2008). Its exclusion was intended to enhance the construct validity and interpretability of the remaining leadership dimensions.

**TABLE 2:** Leadership styles across hierarchical levels.

		*Cronbach’s α*	Hierarchy level			*p*
			Top‐level	Middle‐level	Front‐line	
IC	Individualised consideration	0.710	9.0 (2.0)	9.0 (1.9)	8.3 (1.9)	< 0.001
II	Idealised influence	0.710	9.0 (1.7)	8.9 (1.6)	8.7 (1.6)	0.007
IM	Inspirational motivation	0.660	9.4 (1.6)	9.1 (1.6)	8.8 (1.7)	0.001
IS	Intellectual stimulation	0.670	9.0 (1.8)	8.8 (1.7)	8.4 (1.7)	< 0.001
CR	Contingent reward	0.600	9.9 (1.5)	9.2 (1.7)	9.0 (1.7)	< 0.001
ME	Management‐by‐exception	0.310	9.0 (1.7)	9.0 (1.6)	8.7 (1.6)	0.067
LF	Laissez‐faire leadership	0.820	6.0 (2.8)	6.4 (2.4)	6.5 (2.2)	0.072
Theoretical framework						
Transformational leadership (IC, II, IM, IS)		0.870	9.1 (1.5)	8.9 (1.4)	8.6 (1.4)	< 0.001
Transactional leadership (CR)		0.600	9.9 (1.5)	9.2 (1.7)	9.0 (1.7)	< 0.001
Passive leadership (LF)		0.820	6.0 (2.8)	6.4 (2.4)	6.5 (2.2)	0.072

The three leadership scales were positively correlated with one another. Transformational and transactional leadership were both positively associated with emotional intelligence, indicating that higher levels of emotional intelligence are linked to greater engagement in active leadership behaviours. Age was not significantly associated with emotional intelligence or any leadership style. Professional experience was not associated with emotional intelligence or passive leadership but showed small positive correlations with transformational and transactional leadership (Table 3).

**TABLE 3:** Pearson correlations among leadership styles, emotional intelligence, and demographic variables.

	Transactional leadership	Passive leadership	Emotional intelligence	Years in profession	Age
Transformational leadership	0.667**	0.236**	0.435**	0.090**	0.051
Transactional leadership		0.171**	0.292**	0.079*	0.059
Passive leadership			−0.095**	−0.009	−0.003
Emotional intelligence				0.033	0.000
Years in profession					0.856**

** Significant at 0.01 level.

* Significant at 0.05 level.

### 3.3 Differences in Managers' Leadership Profiles

Mauchly's test of sphericity indicated that the assumption of sphericity was violated (*W* = 0.544, *χ* ^2^(2) = 767.2, *p* < 0.001). Therefore, the Greenhouse–Geisser correction was applied to adjust the degrees of freedom of the F statistic. The repeated measures ANOVA revealed a statistically significant overall difference across the three leadership styles (*F*(1.37, 1703.99) = 878.58, *p* < 0.001).

Post hoc paired‐samples *t*‐tests indicated that transactional leadership scores were significantly higher than transformational leadership scores (*t* (1242) = 11.67, *p* < 0.001), while transformational leadership scores were significantly higher than laissez‐faire leadership scores (*t* (1242) = 33.83, *p* < 0.001). This pattern suggests a general tendency toward more active leadership styles among nurse managers.

A significant interaction between leadership style and hierarchical position was also observed (*F*(2.75, 1703.99) = 12.57, *p* < 0.001). Specifically, transformational and transactional leadership scores increased with higher hierarchical levels, whereas laissez‐faire leadership showed the opposite pattern, being more pronounced among front‐line managers (Figure 1).

**FIGURE 1:**
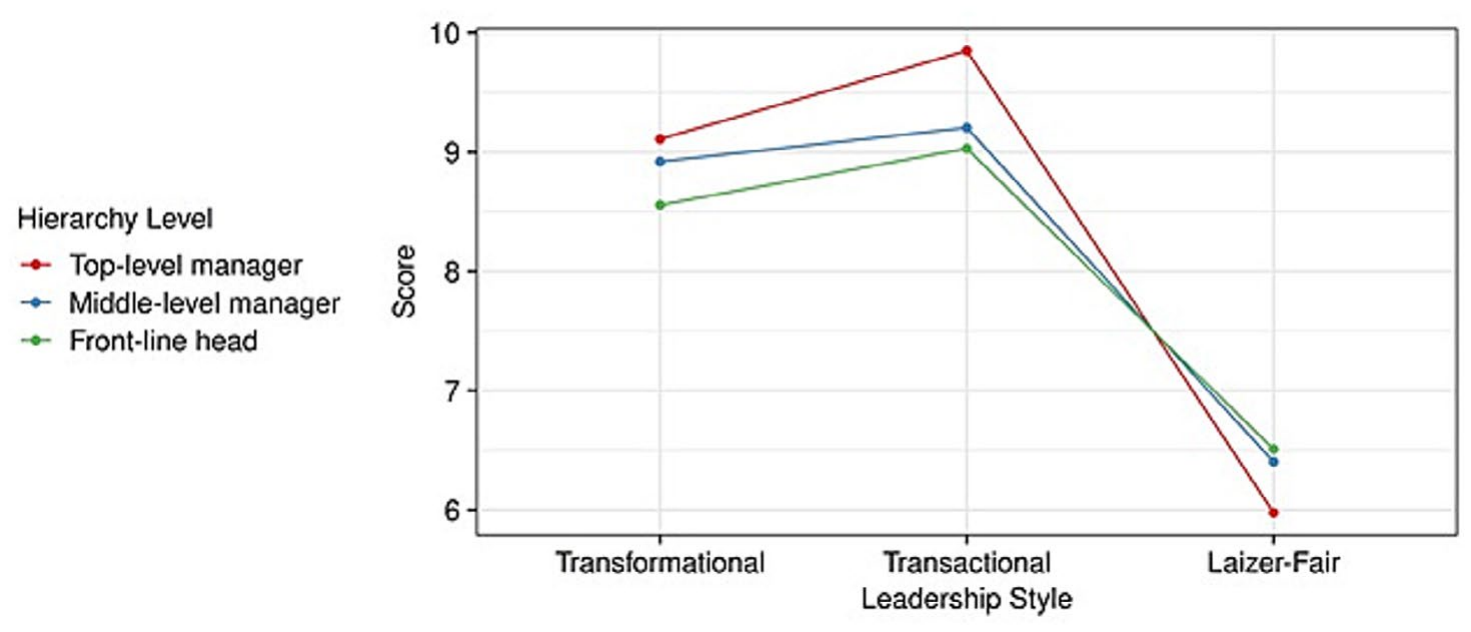
Interaction of hierarchy level and leadership style.

### 3.4 Moderation Analysis Results

The results of the conditional process model are presented in Table 4. Nurse managers working in larger healthcare facilities demonstrated higher levels of emotional intelligence (*B* = 0.184, *p* < 0.001) and lower levels of transactional (*B* = −0.212, *p* = 0.020) and passive leadership (*B* = −0.499, *p* < 0.001), compared with those in smaller facilities.

**TABLE 4:** Moderated mediation model results.

	Emotional intelligence^a^				Transformational leadership^b^				Transactional leadership^c^				Passive leadership^d^			
	*B*	*p*	95% CI		*B*	*p*	95% CI		*B*	*p*	95% CI		*B*	*p*	95% CI	
			Lower	Upper			Lower	Upper			Lower	Upper			Lower	Upper
Constant	5.216	< 0.001	4.993	5.440	4.261	< 0.001	3.372	5.151	5.515	< 0.001	4.396	6.634	7.985	< 0.001	6.326	9.645
Professional experience	**−0.009**	**0.029**	**−0.017**	**−0.001**	**0.017**	**0.040**	**0.001**	**0.032**	0.015	0.126	−0.004	0.035	0.000	0.999	−0.029	0.029
Hierarchy level																
Middle‐level vs. front‐line managers	−0.285	0.117	−0.643	0.072	−0.597	0.379	−1.926	0.733	−0.347	0.684	−2.019	1.326	0.423	0.738	−2.057	2.903
Top‐level vs. front‐line managers	0.089	0.791	−0.571	0.749	−2.029	0.090	−4.375	0.318	−0.673	0.655	−3.625	2.279	−4.348	0.052	−8.724	0.029
Facility size	**0.184**	**< 0.001**	**0.111**	**0.258**	−0.129	0.076	−0.272	0.014	**−0.212**	**0.020**	**−0.392**	**−0.033**	**−0.499**	**< 0.001**	**−0.766**	**−0.233**
Gender	−0.092	0.082	−0.196	0.012	0.103	0.313	−0.097	0.302	−0.175	0.172	−0.425	0.076	0.062	0.742	−0.309	0.434
Hierarchy level × professional experience																
Middle‐level × professional experience	**0.015**	**0.021**	**0.002**	**0.027**	−0.017	0.161	−0.040	0.007	−0.016	0.300	−0.045	0.014	0.004	0.859	−0.040	0.048
Top‐level^a^ × professional experience	0.007	0.513	−0.014	0.027	0.021	0.293	−0.018	0.060	0.016	0.522	−0.033	0.065	0.072	0.053	−0.001	0.145
Emotional intelligence					**0.773**	**< 0.001**	**0.624**	**0.923**	**0.641**	< 0.001	**0.453**	**0.829**	−0.242	0.089	−0.520	0.037
Hierarchy level × emotional intelligence																
Middle‐level × emotional intelligence					**0.254**	**0.026**	**0.030**	**0.479**	0.167	0.246	−0.115	0.449	−0.119	0.576	−0.537	0.299
Top‐level^a^ × emotional intelligence					0.302	0.112	−0.070	0.674	0.134	0.576	−0.334	0.602	0.282	0.426	−0.412	0.976

Indirect effect of professional experience (bootstrap analysis)	Effect	95% CI		Effect	95% CI		Effect	95% CI	
		Lower	Upper		Lower	Upper		Lower	Upper
Top‐level	−0.002	−0.023	0.016	−0.002	−0.018	0.011	−0.000	−0.009	0.007
Middle‐level	0.006	−0.004	0.015	0.004	−0.003	0.012	−0.002	−0.007	0.001
Front‐line	**−0.007**	**−0.015**	**−0.000**	**−0.006**	**−0.012**	**−0.000**	0.002	−0.000	0.007

*Note:* (a) *R* ^2^ = 0.038, *F*(7, 1235) = 6.930, *p* < 0.001. (b) *R* ^2^ = 0.207, *F*(10, 1232) = 32.075, *p* < 0.001. (c) *R* ^2^ = 0.107, *F*(10, 1232) = 14.798, *p* < 0.001. (d) *R* ^2^ = 0.027, *F*(10, 1232) = 3.422, *p* < 0.001. Bold values indicate statistically significant estimates (*p* < 0.05). For bootstrap indirect effects, bold values indicate that the 95% confidence interval does not include zero.

A small but statistically significant positive direct effect of professional experience on transformational leadership was observed (*B* = 0.017, *p* = 0.040). However, conditional indirect effects indicated that, among front‐line managers, professional experience was associated with slight reductions in both transformational and transactional leadership through emotional intelligence (*B* = −0.007 and *B* = −0.006, respectively). These findings suggest that the relationship between experience and leadership is not uniform, but depends on hierarchical context.

The association between professional experience and emotional intelligence varied across hierarchical levels. Among middle‐level managers, professional experience was positively associated with emotional intelligence (*B* = 0.015, *p* = 0.021), whereas among front‐line managers, a negative association was observed (*B* = −0.009, 95% CI = −0.017 to −0.001, *p* = 0.029). The interaction between hierarchy and professional experience was modest in magnitude, accounting for a small proportion of variance in emotional intelligence (Δ*R* ^2^ ≈ 0.004; *F*(2, 1235) = 2.685, *p* = 0.069). Accordingly, these interaction effects should be interpreted as modest contextual influences rather than strong explanatory mechanisms.

Emotional intelligence demonstrated a strong positive association with both transformational (*B* = 0.773, *p* < 0.001) and transactional leadership (*B* = 0.641, *p* < 0.001). In addition, the effect of emotional intelligence on transformational leadership was significantly moderated by hierarchical level, with a stronger effect observed among middle‐level managers (*B* = 0.254, *p* = 0.026). Although this interaction effect was statistically significant, its contribution to explained variance was limited (Δ*R* ^2^ ≈ 0.004; *F*(2, 1232) = 3.023, *p* = 0.049).

Overall, these findings indicate that emotional intelligence is consistently associated with proactive leadership styles, but that its developmental antecedents and its translation into leadership behaviour vary across hierarchical levels. As illustrated in Figure 2, the association between professional experience and emotional intelligence differed across hierarchical levels, with opposite patterns observed among front‐line and middle‐level managers. Notably, the positive association between emotional intelligence and transformational leadership was observed across all hierarchical groups (Figure 3), albeit with differing magnitudes.

**FIGURE 2:**
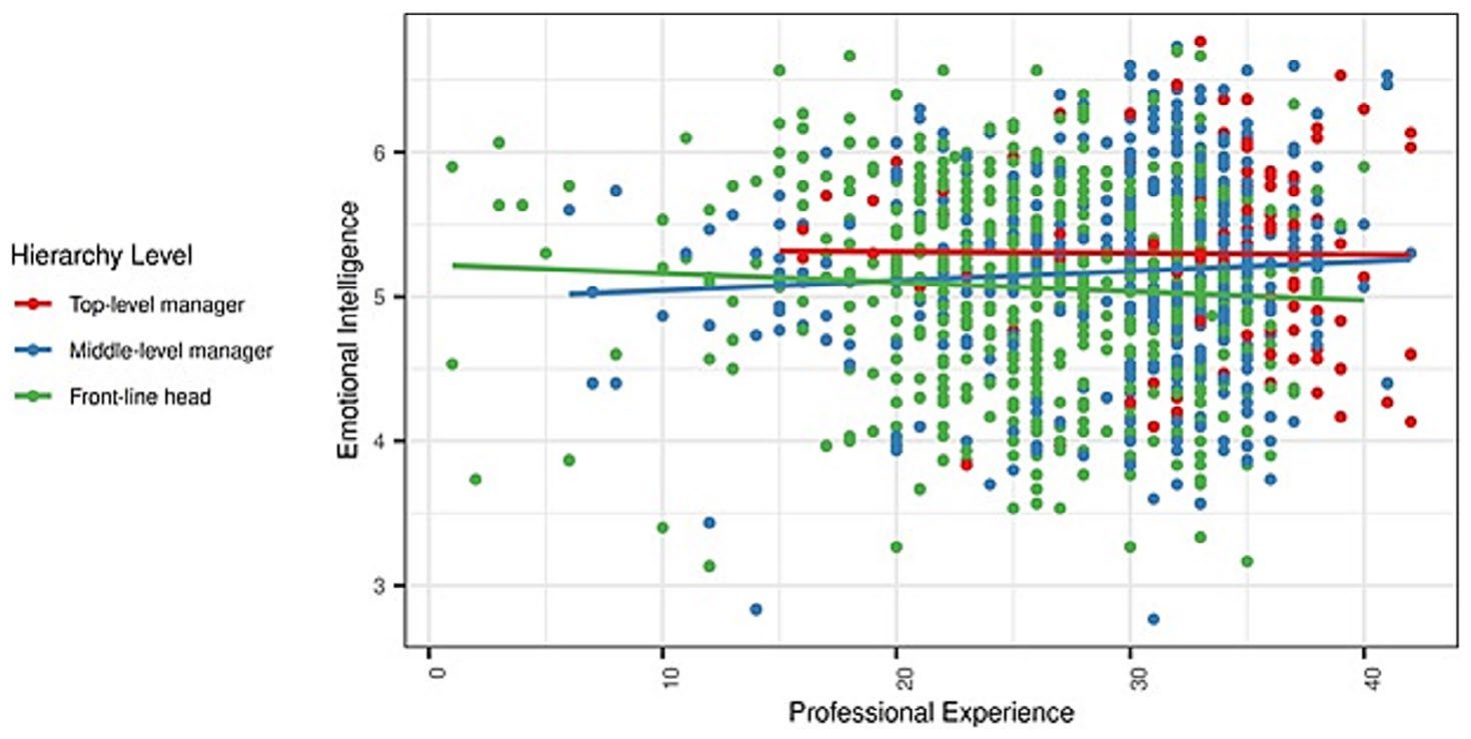
Interaction of hierarchy level and professional experience on emotional intelligence.

**FIGURE 3:**
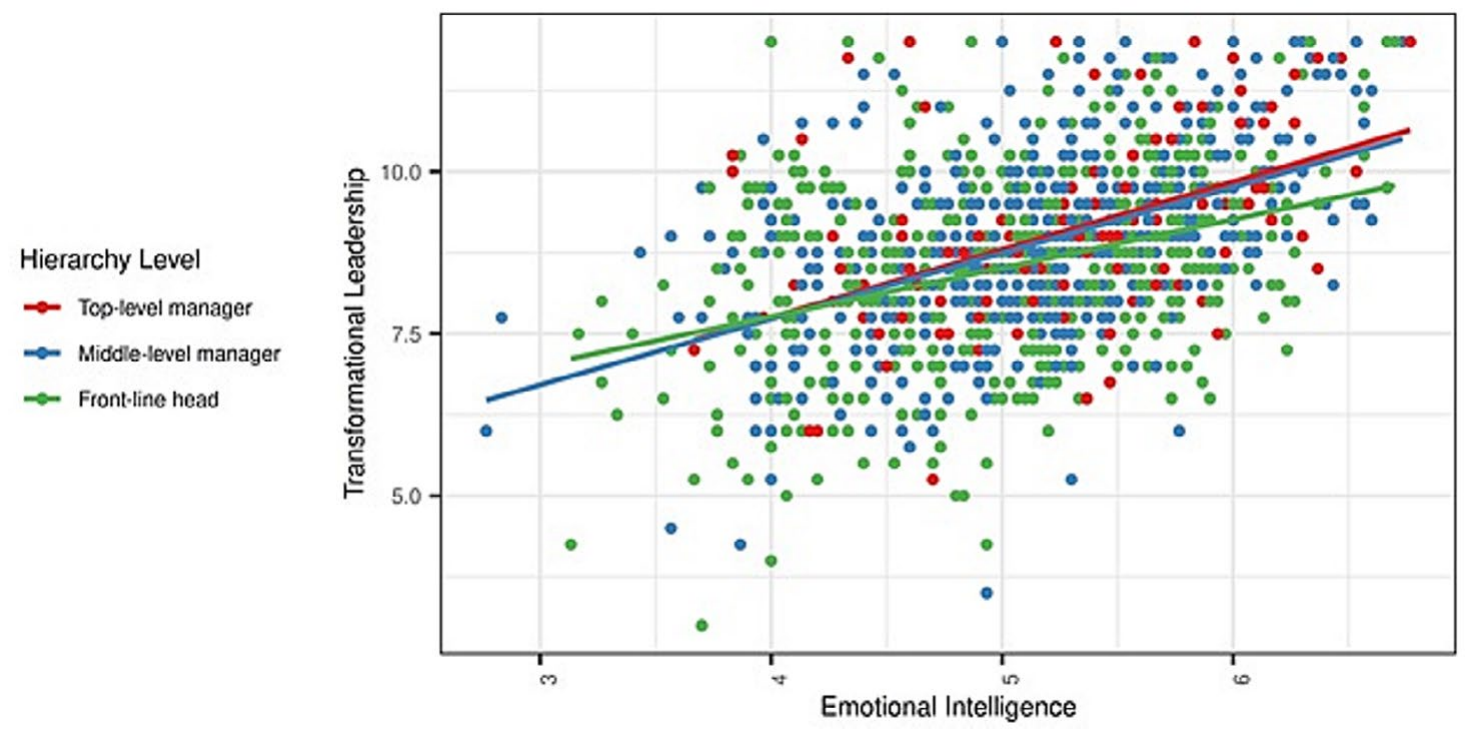
Interaction of hierarchy level and emotional intelligence on transformational leadership.

Overall, the findings provided support for the proposed associations between emotional intelligence and leadership style, while several moderation and conditional process hypotheses demonstrated context‐dependent effects that varied across hierarchical levels.

## 4 Discussion

The present study demonstrates that leadership style among nurse managers is shaped by the interaction of emotional competencies, professional experience, hierarchical position, and organisational context. Transformational and transactional leadership were more pronounced among top‐ and middle‐level managers, whereas front‐line managers exhibited comparatively higher levels of passive leadership. This pattern supports the view that leadership behaviours are role‐embedded, reflecting differences in responsibility, autonomy, and emotional demands across hierarchical levels (Wong et al. 2013; Stordeur et al. 2000).

Emotional intelligence (EI) was positively associated with both transformational and transactional leadership, and negatively associated with passive leadership, reinforcing prior evidence that emotionally competent leaders are better able to mobilise shared purpose, regulate interpersonal dynamics, and adapt leadership behaviours to situational demands (Gardner and Stough 2002; Spano‐Szekely et al. 2016). These findings suggest that EI does not map onto a single leadership style but rather underpins adaptive leadership across relational and task‐oriented domains.

A central contribution of this study is the identification of hierarchy as a conditioning factor in the relationship between EI and leadership. The effect of EI on transformational leadership was strongest among middle‐level managers and weakest among front‐line managers. This finding indicates that the translation of emotional competencies into leadership practice is not uniform, but contingent upon role‐specific conditions. Front‐line managers operate at the intersection of direct patient care and administrative responsibility, often under conditions of time pressure, emotional exposure, staffing constraints, and clinical urgency (Cranage and Foster 2022; Skogsberg et al. 2022). Such sustained exposure to emotionally demanding clinical environments may also increase vulnerability to compassion fatigue and emotional exhaustion, particularly in high‐intensity healthcare settings (Alharbi et al. 2020). Such conditions may restrict opportunities for reflective engagement and limit the enactment of transformational leadership, even in the presence of emotional competence. In this context, passive leadership may reflect role strain and emotional depletion rather than a lack of ability, aligning with evidence linking emotional labour to exhaustion and reduced interpersonal responsiveness (MacCann et al. 2022; Joseph and Newman 2010). This interpretation is consistent with evidence linking demanding work conditions and insufficient organisational support to burnout among nurses and healthcare leaders (Mudallal et al. 2017).

The differential association between professional experience and EI across hierarchical levels further supports this interpretation. While experience was positively associated with EI among middle‐level managers, it was negatively associated among front‐line managers. This pattern suggests that prolonged exposure to emotionally demanding environments without adequate organisational support may erode, rather than enhance, emotional capacities over time (Zeidner et al. 2009; Cherry et al. 2014). The availability of psychological resilience resources and social support may therefore play an important role in moderating the long‐term emotional impact of leadership strain in healthcare settings (Labrague 2021). Thus, the developmental trajectory of EI appears to depend not only on exposure, but on the structural conditions under which such exposure occurs.

In addition, managers in larger healthcare facilities reported higher EI and lower passive leadership. Although larger organisations may provide greater access to interdisciplinary collaboration, managerial training, and formalised support structures, which could buffer emotional strain and reinforce proactive leadership behaviours (Marx 2017; Niinihuhta and Häggman‐Laitila 2022), these interpretations remain theoretical and were not directly examined in the present study. Other contextual factors, including organisational culture, staffing conditions, institutional climate, or urban–regional differences, may also contribute to the observed associations. Conversely, smaller or more fragmented organisational environments may constrain both the development and the expression of emotionally intelligent leadership.

Taken together, these findings indicate that emotional intelligence operates not merely as an individual resource, but as a structurally conditioned capacity, enabled or constrained by organisational context and hierarchical positioning. Leadership development in nursing should therefore extend beyond individual skill acquisition to include structural supports, such as protected supervision time, formal mentorship, reflective practice opportunities, and manageable workload conditions (Specchia et al. 2021). These organisational supports may additionally strengthen resilience and emotional sustainability among nurse managers working in high‐demand clinical environments (Wei et al. 2019). Supporting the emotional labour of front‐line managers is not supplementary, but foundational to sustainable and effective leadership.

Although statistically significant interaction effects were identified, their magnitude was relatively modest. However, such effect sizes are typical in organisational and behavioural research, where outcomes are shaped by multiple interacting factors. Although these interaction effects were relatively small, such findings may still be meaningful within complex organisational environments, where leadership behaviours are influenced by multiple interacting structural and interpersonal factors rather than single dominant predictors.

This study advances current knowledge by demonstrating that the expression of emotionally intelligent leadership is contingent upon organisational context. Leadership capacity is not solely a function of individual competence; rather, it emerges within—and is shaped by—the conditions under which managerial work is performed. Recognising emotional labour as a structural component of nursing management is therefore critical for informing leadership development strategies and organisational design.

Importantly, the contribution of this study lies not in establishing the association between emotional intelligence and leadership, but in demonstrating that this relationship is structurally conditioned. Emotional intelligence does not operate uniformly; its development and expression depend on hierarchical positioning and organisational context. By shaping leadership behaviours, emotional intelligence may indirectly influence team functioning and, ultimately, the quality and continuity of patient care. Given the cross‐sectional design, the observed mediation effects should be interpreted as statistical associations consistent with indirect pathways, rather than evidence of causal mechanisms.

## 5 Limitations of the Study

This study should be interpreted in light of several limitations. First, the research was conducted within a specific cultural and healthcare system context. The sample was drawn from public healthcare facilities, where hierarchical structures, organisational dynamics, and the experience of emotional labour may differ from those in other healthcare systems. Accordingly, caution is warranted when generalising these findings beyond comparable institutional and cultural settings. Future research could examine whether the observed hierarchical patterns are replicated across different national and organisational contexts.

Second, the cross‐sectional design precludes causal inferences regarding the relationships between emotional intelligence, professional experience, and leadership style. Although the analytical model tested conditional and indirect pathways, the directionality of these associations cannot be definitively established. Longitudinal or prospective designs would be required to clarify developmental trajectories and causal mechanisms.

Third, the interaction effects observed were modest in magnitude, indicating that the model provides a partial account of leadership processes in nursing management.

All variables were assessed through self‐report measures, which may introduce common method bias and increase the likelihood of inflated associations due to shared method variance. In addition, leadership behaviours were evaluated exclusively from the perspective of the managers themselves, rather than through subordinate or multi‐source assessments. Future studies could strengthen validity by incorporating peer evaluations, staff ratings, observational approaches, or longitudinal designs.

Finally, the analytical approach assumes linear relationships between variables, which may not fully reflect the dynamic and potentially non‐linear nature of emotional and leadership processes. Future studies may benefit from exploring non‐linear or multilevel modelling approaches, as well as incorporating additional contextual variables, such as workload intensity, organisational climate, or support structures.

## 6 Conclusion

This study highlights the interdependence of emotional, experiential, and structural factors in shaping leadership styles among nurse managers. Emotional intelligence emerged as a key mechanism through which professional experience may be associated with leadership style; however, this relationship was conditional and varied across hierarchical levels. Front‐line managers, positioned at the interface of clinical and managerial demands, appeared particularly vulnerable to emotional strain, which may constrain the enactment of proactive leadership behaviours.

These findings underscore that emotional intelligence should not be conceptualised solely as an individual competency, but as a capacity that is contextually supported or constrained. Organisational structures, workload conditions, and access to reflective and supervisory resources play a critical role in enabling nurse managers to enact emotionally attuned leadership. Furthermore, the association between larger organisational settings and higher EI suggests that institutional scale and resource availability may shape leadership environments in meaningful ways.

Strengthening leadership in nursing therefore requires a dual focus: enhancing individual emotional competencies while simultaneously addressing the structural conditions that shape their expression. Sustainable leadership development may therefore require organisational strategies that actively support professional wellbeing, emotional regulation, and workforce engagement (Shanafelt and Noseworthy 2017). Interventions such as structured mentorship, protected time for reflective supervision, and organisational practices that recognise emotional labour are essential. In particular, supporting front‐line managers is crucial for sustaining the quality, continuity, and humanity of care within contemporary healthcare systems.

## Funding

The authors have nothing to report.

## Ethics Statement

The study was conducted in accordance with institutional guidelines for research involving human participants. Ethics approval was obtained from the Research Ethics and Deontology Committee (Approval No: 59/2024; Meeting 4/27‐05‐2024; Decision date: 15/07/2024).

## Consent

Informed consent was obtained from all participants.

## Conflicts of Interest

The authors declare no conflicts of interest.

## Data Availability

The data that support the findings of this study are available on request from the corresponding author. The data are not publicly available due to privacy or ethical restrictions.

## References

[jan70680-bib-0002] Alharbi, J. , D. Jackson , and K. Usher . 2020. “The Potential for COVID‐19 to Contribute to Compassion Fatigue in Critical Care Nurses.” Journal of Clinical Nursing 29, no. 15–16: 2762–2764. 10.1111/jocn.15314. PMC7267232 32344460

[jan70680-bib-0003] Bakker, A. B. , and E. Demerouti . 2007. “The Job Demands‐Resources Model: State of the Art.” Journal of Managerial Psychology 22, no. 3: 309–328. 10.1108/02683940710733115.

[jan70680-bib-0005] Bass, B. M. , and B. J. Avolio . 1990. Transformational Leadership Development: Manual for the Multifactor Leadership Questionnaire. Consulting Psychologists Press.

[jan70680-bib-0006] Bass, B. M. , and R. E. Riggio . 2006. Transformational Leadership. Psychology Press.

[jan70680-bib-0007] Boamah, S. A. , H. K. S. Laschinger , C. Wong , and S. Clarke . 2018. “Effect of Transformational Leadership on Job Satisfaction and Patient Safety Outcomes.” Nursing Outlook 66, no. 2: 180–189. 10.1016/j.outlook.2017.10.004. 29174629

[jan70680-bib-0011] Cherry, M. G. , I. Fletcher , H. O'Sullivan , and T. Dornan . 2014. “Emotional Intelligence in Medical Education: A Critical Review.” Medical Education 48, no. 5: 468–478. 10.1111/medu.12406. 24712932

[jan70680-bib-0013] Cranage, K. , and K. Foster . 2022. “Mental Health Nurses' Experience of Challenging Workplace Situations: A Qualitative Descriptive Study.” International Journal of Mental Health Nursing 31, no. 3: 665–676. 10.1111/inm.12986. PMC9314796 35347822

[jan70680-bib-0015] Cummings, G. G. , K. Tate , S. Lee , et al. 2018. “Leadership Styles and Outcome Patterns for the Nursing Workforce and Work Environment: A Systematic Review.” International Journal of Nursing Studies 85: 19–60. 10.1016/j.ijnurstu.2018.04.016. 29807190

[jan70680-bib-0018] Gardner, L. , and C. Stough . 2002. “Examining the Relationship Between Leadership and Emotional Intelligence in Senior Level Managers.” Leadership and Organization Development Journal 23, no. 2: 68–78. 10.1108/01437730210419198.

[jan70680-bib-0019] Goleman, D. 1998. “What Makes a Leader?” Harvard Business Review 76, no. 6: 93–102. 10187249

[jan70680-bib-0021] Harms, P. D. , and M. Credé . 2010. “Emotional Intelligence and Transformational and Transactional Leadership: A Meta‐Analysis.” Journal of Leadership and Organizational Studies 17, no. 1: 5–17. 10.1177/1548051809350894.

[jan70680-bib-0023] Hayes, A. F. 2017. Introduction to Mediation, Moderation, and Conditional Process Analysis: A Regression‐Based Approach. Guilford Publications.

[jan70680-bib-0024] Heller, S. , J. Ullrich , and M. S. Mast . 2023. “Power at Work: Linking Objective Power to Psychological Power.” Journal of Applied Social Psychology 53: 5–20. 10.1111/jasp.12922.

[jan70680-bib-0025] Hinkin, T. R. , and C. A. Schriesheim . 2008. “An Examination of ‘Nonleadership’: From Laissez‐Faire Leadership to Leader Reward Omission and Punishment Omission.” Journal of Applied Psychology 93, no. 6: 1234–1248. 10.1037/a0012875. 19025245

[jan70680-bib-0026] Joseph, D. L. , and D. A. Newman . 2010. “Emotional Intelligence: An Integrative Meta‐Analysis and Cascading Model.” Journal of Applied Psychology 95: 54–78. 10.1037/a0017286. 20085406

[jan70680-bib-0027] Kahn, R. L. , D. M. Wolfe , R. P. Quinn , J. D. Snoek , and R. A. Rosenthal. 1964. Organizational Stress: Studies in Role Conflict and Ambiguity. John Wiley.

[jan70680-bib-0029] Labrague, L. J. 2021. “Psychological Resilience, Coping Behaviours and Social Support Among Health Care Workers During the COVID‐19 Pandemic: A Systematic Review of Quantitative Studies.” Journal of Nursing Management 29, no. 7: 1893–1905. 10.1111/jonm.13336. PMC8250179 33843087

[jan70680-bib-0031] MacCann, C. , K. S. Double , and I. E. Clarke . 2022. “Lower Avoidant Coping Mediates the Relationship of Emotional Intelligence With Well‐Being and Ill‐Being.” Frontiers in Psychology 13: 835819. 10.3389/fpsyg.2022.835819. PMC9396212 36017435

[jan70680-bib-0033] Marx, T. G. 2017. “The Impacts of Company Size on Leadership.” Management and Organizational Studies 4, no. 1: 82. 10.5430/MOS.V4N1P82.

[jan70680-bib-0035] Montgomery, A. , F. Spânu , A. Băban , and E. Panagopoulou . 2015. “Job Demands, Burnout, and Engagement Among Nurses: A Multi‐Level Analysis of ORCAB Data Investigating the Moderating Effect of Teamwork.” Burnout Research 2, no. 2–3: 71–79. 10.1016/j.burn.2015.06.001. PMC4710673 26877971

[jan70680-bib-0036] Mudallal, R. H. , W. A. M. Othman , and N. F. Al Hassan . 2017. “Nurses' Burnout: The Influence of Leader Empowering Behaviors, Work Conditions, and Demographic Traits.” Inquiry: The Journal of Health Care Organization, Provision, and Financing 54: 0046958017724944. 10.1177/0046958017724944. PMC5798741 28844166

[jan70680-bib-0039] Niinihuhta, M. , and A. Häggman‐Laitila . 2022. “A Systematic Review of the Relationships Between Nurse Leaders' Leadership Styles and Nurses' Work‐Related Well‐Being.” International Journal of Nursing Practice 28, no. 5: e13040. 10.1111/ijn.13040. PMC9788052 35102648

[jan70680-bib-0041] Prezerakos, P. E. 2018. “Nurse Managers' Emotional Intelligence and Effective Leadership: A Review of the Current Evidence.” Open Nursing Journal 12: 86–92. 10.2174/1874434601812010086. PMC5997858 29997711

[jan70680-bib-0042] R Core Team . 2021. R: A Language and Environment for Statistical Computing. R Foundation for Statistical Computing.

[jan70680-bib-0045] Shanafelt, T. D. , and J. H. Noseworthy . 2017. “Executive Leadership and Physician Well‐Being: Nine Organizational Strategies to Promote Engagement and Reduce Burnout.” In Mayo Clinic Proceedings, vol. 92, no. 1, 129–146. Elsevier. 10.1016/j.mayocp.2016.10.004. 27871627

[jan70680-bib-0048] Skogsberg, M. , G. Jarl , and M. Matérne . 2022. “Health Care Workers' Need for Support From Managers During the Initial Phase of the COVID‐19 Pandemic.” BMC Health Services Research 22, no. 1: 1563. 10.1186/s12913-022-08937-9. PMC9768758 36544137

[jan70680-bib-0049] Spano‐Szekely, L. , M. T. Quinn Griffin , J. Clavelle , and J. J. Fitzpatrick . 2016. “Emotional Intelligence and Transformational Leadership in Nurse Managers.” Journal of Nursing Administration 46, no. 2: 101–108. 10.1097/NNA.0000000000000303. 26796823

[jan70680-bib-0050] Specchia, M. L. , M. R. Cozzolino , E. Carini , et al. 2021. “Leadership Styles and Nurses' Job Satisfaction. Results of a Systematic Review.” International Journal of Environmental Research and Public Health 18, no. 4: 1552. 10.3390/ijerph18041552. PMC7915070 33562016

[jan70680-bib-0051] Stamatopoulou, M. , P. Galanis , and P. Prezerakos . 2016. “Psychometric Properties of the Greek Translation of the Trait Emotional Intelligence Questionnaire‐Short Form (TEIQue‐SF).” Personality and Individual Differences 95: 80–84. 10.1016/j.paid.2016.02.035.

[jan70680-bib-0052] Stordeur, S. , C. Vandenberghe , and W. D'hoore . 2000. “Leadership Styles Across Hierarchical Levels in Nursing Departments.” Nursing Research 49, no. 1: 37–43. 10.1097/00006199-200001000-00006. 10667627

[jan70680-bib-0054] Tyczkowski, B. , C. Vandenhouten , J. Reilly , G. Bansal , S. M. Kubsch , and R. Jakkola . 2015. “Emotional Intelligence (EI) and Nursing Leadership Styles Among Nurse Managers.” Nursing Administration Quarterly 39, no. 2: 172–180. 10.1097/NAQ.0000000000000094. 25714956

[jan70680-bib-0056] Wei, H. , P. Roberts , J. Strickler , and R. W. Corbett . 2019. “Nurse Leaders' Strategies to Foster Nurse Resilience.” Journal of Nursing Management 27, no. 4: 681–687. 10.1111/jonm.12736. 30449038

[jan70680-bib-0058] Wong, C. A. , G. G. Cummings , and L. Ducharme . 2013. “The Relationship Between Nursing Leadership and Patient Outcomes: A Systematic Review Update.” Journal of Nursing Management 21, no. 5: 709–724. 10.1111/jonm.12116. 23865924

[jan70680-bib-0060] Zeidner, M. , G. Matthews , and R. D. Roberts . 2009. What We Know About Emotional Intelligence: How It Affects Learning, Work, Relationships, and Our Mental Health. MIT Press.

